# Myxome géant de l'oreillette gauche appendu à la valve mitrale hyper mobile symptomatique: à propos d'un cas

**Published:** 2012-09-30

**Authors:** Euloge Yiagnigni Mfopou, Pierre Ndobo, Frank Daniel Zouna Ngouagna, Olivier Pancha Mbouembouo

**Affiliations:** 1 Hôpital Central de Yaoundé, Yaoundé, Cameroun; 2 Hôpital de N'Gaoundéré, N'Gaoundéré, Cameroun

**Keywords:** Myxome, oreillette gauche, echocardiographie, myxoma, left atrium, echocardiography

## Abstract

Nous rapportons le cas d'un myxome de l'oreillette gauche chez un patient âgé de 28 ans, reçu en consultation externe pour douleur précordiale à l'effort et lipothymie. L’échocardiographie a permis de poser le diagnostic de myxome géant hyper mobile avec impact hémodynamique. La prise en charge a été limite à cause du plateau technique très réduite.

## Introduction

Les myxomes cardiaques sont les tumeurs primitives intracardiaques les plus fréquentes de l'adulte avec une fréquence de 0.5 par million d'habitants par an [1]. Ils représentent 50% des tumeurs cardiaques. Quoique bénin d′un point de vue histologique, sa localisation peut mettre en jeu le pronostic vital du patient. Sa symptomatologie insidieuse en rend souvent difficile le diagnostic jusqu'au stade de complications. Environ 50% des patients porteurs de myxomes présentent des symptômes dus à une embolie cérébrale ou périphérique, ou à une obstruction intracardiaque. Dix pour cent sont complètement asymptomatiques. L’échocardiographie transthoracique et transæsophagienne permettent le diagnostic avec une sensibilité proche de 100%.

## Observation

Un jeune homme de 28 ans, reçu en consultation externe pour douleurs précordiales à l'effort à type d'angine de poitrine associées à une sensation de vertiges, d’étourdissement obligeant l'arrêt de l'activité et évoluant depuis près de six mois. L'interrogatoire exclut une notion d'angine de gorge et de facteurs de risque cardiovasculaire.

L'examen physique révèle un bon état général, la tension artérielle est de 123/84 mmHg (en position couchée) et 94/70 mmHg (en position debout). La fréquence du pouls radial est estimée à 80 battements par minute en position couchée et 66 battements par minute en position debout. Les conjonctives sont rosées. La bouche et la dentition sont normales. L'auscultation pulmonaire révèle de discrets râles crépitant aux bases pulmonaires. L'auscultation cardiaque révèle un souffle mitral, pan systolique, aigu, d'intensité 3/6, à type de jet de vapeur et irradiant à l'aisselle gauche; Un roulement diastolique au foyer mitral, d'intensité 3-4/6. Le reste de l'examen physique est sans particularités. L’électrocardiogramme a montré un rythme sinusal, régulier avec une fréquence cardiaque de 75 / min. On note une hypertrophie atriale gauche et une absence de signes d'ischémie ou de lésions.

**Figure 1:**
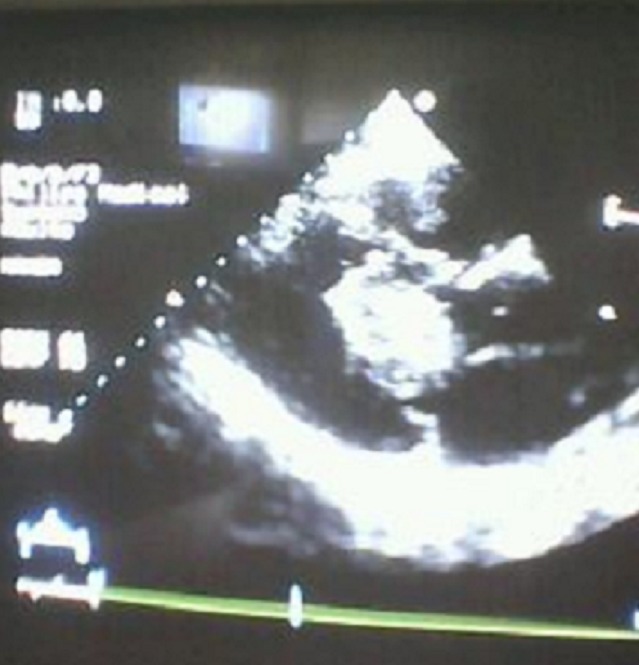
Vue apicale quatre cavités myxome en systole

**Figure 2:**
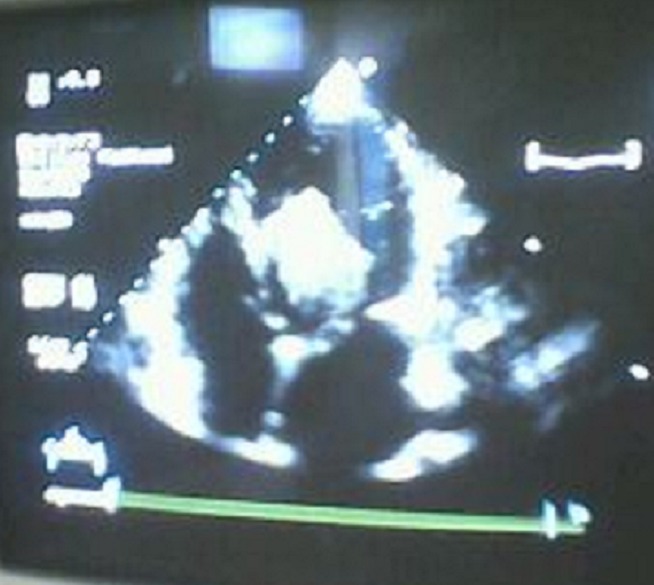
Vue apical quatre cavités myxome en diastole

**Figure 3:**
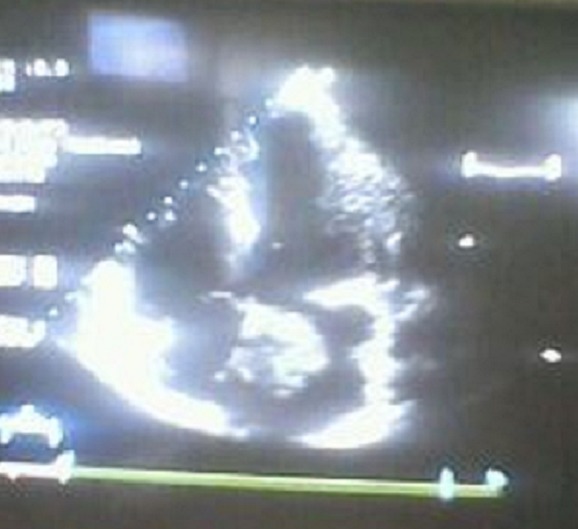
Vue parasternale myxome en diastole

L’échocardiographie montre une oreillette gauche dilatée contenant une masse pédiculée de 16.4 cm^2^, hétérogène à surface irrégulière, appendue à la face atriale de la valve mitrale antérieure et très mobile, traversant l'orifice mitral et se retrouvant presque entièrement dans le ventricule gauche pendant la diastole avec une obstruction subtotale de l'orifice mitral et refoulée dans l'oreillette gauche pendant la systole (Figure 1, Figure 2, Figure 3). Le gradient de pression maximal et moyen au niveau de la valve mitrale étaient respectivement 13.5 mmHg et 7.1 mmHg. Le ventricule gauche avait une fonction systolique normale (Fraction d’éjection de 57%). On a noté une insuffisance mitrale de 1^er^ grade. La pression pulmonaire systolique était élevée à 55 mmHg. Le reste sans particularités. Dans l'attente d'une intervention chirurgicale, nous avons initié une anti-coagulation préventive et recommandé une éviction de tout effort.

## Discussion

Les tumeurs cardiaques primitives représentent moins de 0.2% de l'ensemble des néoplasies, trois quarts de ces tumeurs sont bénignes et la moitié de ces tumeurs sont des myxomes, la femme est plus souvent touchée [2]. Les myxomes peuvent siéger dans les oreillettes, les ventricules ou sur la valve mitrale, avec une nette prédilection pour l'oreillette gauche [3, 4]; tel est le cas de notre patient. La symptomatologie reste variable et dépend de la localisation, la forme, la taille et l'activité du patient [5]. La manifestation courante est le tableau d'insuffisance cardiaque suivie de l'embolisation [5, 6]. Notre patient a présenté à l'examen physique des signes de maladie mitrale et des signes d'insuffisance cardiaque gauche à l'effort (le bas débit cardiaque et la congestion pulmonaire). Par ailleurs la variation hémodynamique en fonction de la position de notre patient ferait penser à une hypotension orthostatatisque; ceci pourrait s'expliquer par la grande mobilité de la masse, dont en position debout l'effet de la pesanteur favoriserait l'obstruction quasi permanente de l'orifice valvulaire induisant la baisse du volume télé diastolique ventriculaire puis du débit cardiaque. Comme dans toute la littérature, l’échocardiographie a permis de diagnostiquer précisément la masse; d'autres méthodes diagnostiques non invasives tels que l'imagerie par résonnance magnétique, la tomographie peuvent être utilisées pour plus de précision diagnostique [4]. L'aspect hyper mobile de la masse avec un impact hémodynamique est une indication très urgente de l'intervention chirurgicale afin d’éviter l'embolisation [7]. Nous n'avons pas pu mettre en exécution cette recommandation au vu du plateau technique chirurgical très limité dans notre milieu.

Cet article est un cas clinique qui peut être considéré comme étant isolé. Toutefois, ce cas clinique ressort la variabilité de la manifestation du myxome et attire l'attention du clinicien à mener toujours des investigations devant toute symptomatologie cardiaque.

## Conclusion

Le myxome, quoique étant une masse bénigne, peut induire une symptomatologie dramatique et mettre en jeu le pronostic vital du patient. En cas de suspicion, Il doit être diagnostiqué précocement et pris en charge notamment par la méthode chirurgicale. Dans notre milieu, même devant les cas comme celui-ci exposant le patient à une complication fatale à type d'obstruction de l'orifice avec chute du débit sanguin, la prise en charge optimale urgente reste hypothétique à cause du plateau technique limité.
